# Growth Factor Signaling in Solid Organ Transplantation: A Conceptual Framework for Chronic Remodeling and Survival

**DOI:** 10.3390/ijms27104542

**Published:** 2026-05-19

**Authors:** Urszula Łacek, Cezary Gaczyński, Małgorzata Goszka, Aleksandra Polikowska, Natalia Serwin, Barbara Dołęgowska, Elżbieta Cecerska-Heryć

**Affiliations:** Department of Laboratory Medicine, Pomeranian Medical University of Szczecin, 70-111 Szczecin, Poland

**Keywords:** TGF-β signaling, PDGF, VEGF-A, IGF-1, transplant immunology, precision medicine

## Abstract

Long-term survival after solid organ transplantation remains limited by chronic remodeling, fibrosis, vascular complications, and malignancy despite advances in immunosuppressive therapy. Current monitoring strategies primarily rely on functional and immunological parameters that often identify complications only after irreversible injury has occurred. There is a critical need for earlier, mechanistically informative biomarkers that can predict survival outcomes. Many platelet-associated growth factors (PDGF, TGF-β, VEGF, EGF, and IGF-1) are stored in platelet α-granules but can also originate from immune, endothelial, and stromal cells, regulate angiogenesis, extracellular matrix deposition, immune modulation, and tissue repair—processes central to graft adaptation and chronic injury. In this review, we propose the growth factor signaling network as a conceptual framework that potentially links platelet biology, ischemia-reperfusion injury, alloimmune responses, and chronic immunosuppression to sustained growth factor signaling and maladaptive graft remodeling. This framework should be interpreted as a biologically plausible integrative model rather than a fully validated mechanistic pathway in transplant recipients. Importantly, direct clinical evidence linking platelet activation markers (e.g., P-selectin, PF4, β-thromboglobulin) with circulating growth factor levels and long-term transplant outcomes remains limited, highlighting a critical gap in current biomarker research. Emerging clinical evidence suggests their potential prognostic relevance in transplant outcomes. Elevated TGF-β levels have been associated with increased risk of opportunistic infections, while early postoperative IGF-1 concentrations predict short-term survival. Increased VEGF-A levels correlate with primary graft dysfunction and cardiac allograft vasculopathy, while PDGF isoforms contribute to fibrotic and vascular progression across transplanted organs. However, their clinical applicability is limited by methodological variability and lack of large-scale validation. Rather than serving solely as markers of rejection, platelet-associated growth factors may reflect dynamic processes involved in transplant remodeling and mortality risk. Incorporating growth factor profiling into multiparametric survival prediction models may improve early risk stratification and support precision post-transplant management strategies.

## 1. Introduction

**Figure 1:**
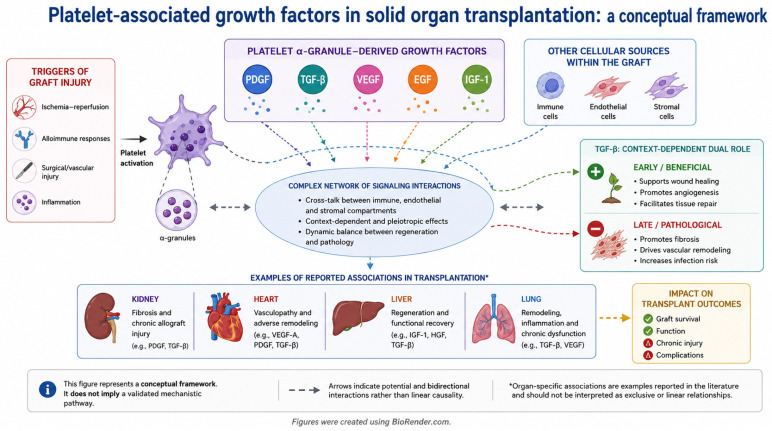
Platelet-associated growth factors in solid organ transplantation: a conceptual framework. Graft injury, ischemia-reperfusion, and alloimmune responses may be associated with platelet activation and release of bioactive mediators from platelet α-granules. These include PDGF, TGF-β, VEGF, EGF, and IGF-1, which regulate interactions between immune, endothelial, and stromal cells, contributing to tissue remodeling, angiogenesis, and inflammation [4,5,6]. Importantly, these growth factors are not exclusively platelet-derived and may also originate from other cellular sources within the graft microenvironment. Rather than representing a single linear pathway, these processes reflect a complex network of signaling interactions influencing transplant outcomes. Organ-specific associations include kidney fibrosis (PDGF), liver function and survival (IGF-1), cardiac vasculopathy (VEGF-A), and lung remodeling or chronic allograft dysfunction (TGF-β/VEGF) [7,8,9,10,11,12,13,14,15,16,17,18,19,20,21,22,23,24,25,26,27,28,29]. The dual role of TGF-β reflects context-dependent effects, with early regenerative and late pathological consequences. This figure represents a conceptual model and does not imply a validated mechanistic pathway. Figures were created using BioRender.com.

Solid organ transplantation remains one of the most effective treatments for end-stage organ failure; however, long-term outcomes are still constrained by chronic graft remodeling, fibrosis, vascular complications, opportunistic infections, and malignancy [1]. Although conventional post-transplant monitoring relies mainly on functional and immunological parameters, these markers often detect injury only after clinically relevant damage has occurred. Growth factors are key regulators of wound healing, angiogenesis, immune modulation, and tissue remodeling [2]. Among them, PDGF, TGF-β, VEGF, EGF, and IGF-1 have attracted increasing interest in transplant medicine due to their associations with fibrosis, endothelial dysfunction, vascular remodeling, and graft survival [3]. These mediators are not exclusively platelet-derived, as they may also be released by macrophages, endothelial cells, fibroblasts, and other cell populations involved in graft injury and repair. In this review, we propose the growth factor signaling network as a conceptual framework integrating platelet biology with broader growth factor signaling in transplantation, while acknowledging that direct clinical evidence linking platelet activation to sustained growth factor release in transplant recipients remains limited. The conceptual framework of platelet-associated growth factors in solid organ transplantation is summarized in Figure 1.

## 2. Growth Factors Involved in Transplant Remodeling

Growth factors such as PDGF, TGF-β, VEGF, IGF-1, and EGF are key regulators of angiogenesis, immune modulation, extracellular matrix remodeling, and tissue repair—processes central to graft adaptation and long-term transplant outcomes [1,2,3,4,5]. Detailed evidence supporting their roles in transplantation is summarized in Table 1.

Although these mediators are enriched in platelet α-granules, they are not exclusively platelet-derived. TGF-β, PDGF, VEGF, and EGF may also be produced by immune cells (e.g., monocytes and macrophages), endothelial cells, fibroblasts, and other stromal components involved in graft injury, inflammation, and repair [31,32,33,34,35]. Therefore, circulating concentrations should be interpreted as a composite signal reflecting multiple cellular sources rather than platelet activation alone.

Direct mechanistic links between platelet activation and circulating growth factor levels in clinical transplant settings remain poorly defined.

In the context of solid organ transplantation, dysregulated growth factor signaling has been implicated in fibrosis, vascular remodeling, endothelial dysfunction, and chronic graft injury [19,39]. These processes provide the biological basis for their potential use as prognostic biomarkers of survival and graft outcomes.

**Figure 2:**
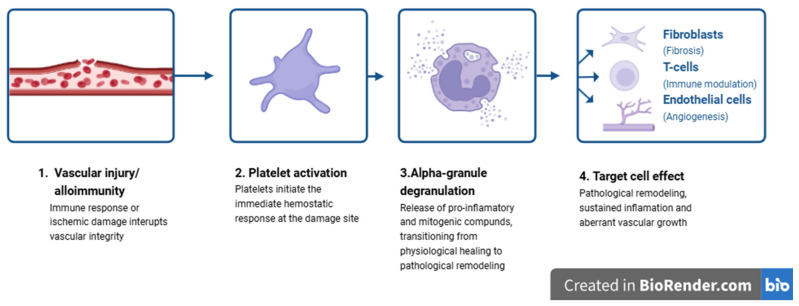
Vascular injury and alloimmune activation may promote platelet activation and the release of bioactive mediators, including PDGF, TGF-β, VEGF, EGF, and IGF-1. These factors regulate fibroblast, immune, and endothelial cell responses, contributing to inflammation, angiogenesis, and extracellular matrix remodeling. Importantly, these mediators are not exclusively platelet-derived and may also originate from immune and stromal cells within the graft microenvironment. While initially supporting tissue repair, sustained signaling is associated with pathological remodeling, fibrosis, and chronic graft dysfunction [31,32,33,34,35]. Figures were created using BioRender.com.

The conceptual relationship between platelet biology, growth factor signaling, and graft remodeling is illustrated in (Figure 2).

### 2.1. Platelet-Derived Growth Factor (PDGF)

PDGF is a key regulator of cell proliferation, migration, and extracellular matrix remodeling, with established roles in tissue repair and vascular remodeling [9,10,11,12]. Although enriched in platelet α-granules, PDGF is also produced by macrophages, endothelial cells, and fibroblasts under conditions of tissue injury and inflammation [31,32,33]. In transplantation, PDGF signaling has been implicated with fibrotic remodeling, vascular proliferation, and chronic graft dysfunction across multiple organs [13,14,15,16].

### 2.2. Epidermal Growth Factor (EGF)

EGF regulates cell proliferation and tissue regeneration via activation of EGFR-dependent signaling pathways, including PI3K/Akt and MAPK/ERK [36]. While it supports epithelial repair under physiological conditions, dysregulated EGF signaling is associated with chronic inflammation and pathological remodeling [37]. In transplantation, its role appears to be context-dependent and less well defined than that of other growth factors.

### 2.3. Insulin-like Growth Factor (IGF)

IGF-1 is a peptide growth factor involved in cell survival, proliferation, and tissue regeneration, with its bioavailability tightly regulated by insulin-like growth factor-binding proteins (IGFBPs) [17,18,19,20].

**Figure 3:**
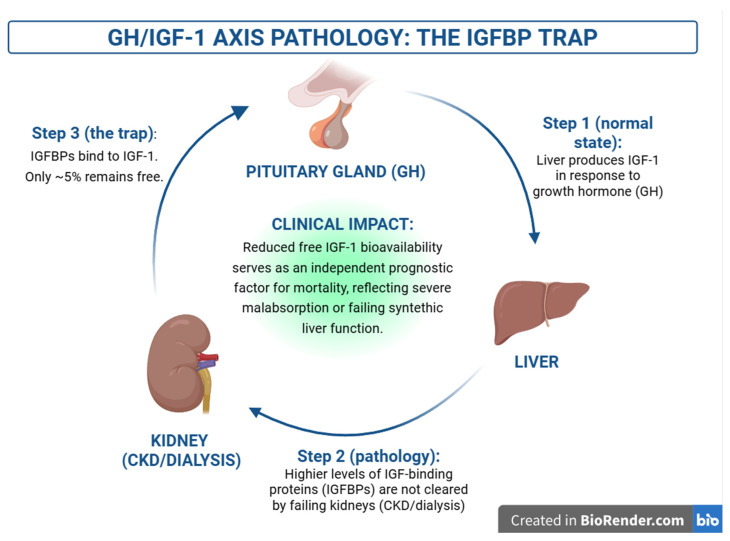
Dysregulation of the GH/IGF-1 axis and the “IGFBP trap” in kidney and liver pathology. Under physiological conditions, growth hormone (GH) stimulates hepatic production of IGF-1, which circulates in both free and protein-bound forms [17,18,19,20]. In pathological states, particularly in chronic kidney disease (CKD) and during renal replacement therapy, impaired renal clearance and increased production of insulin-like growth factor-binding proteins (IGFBPs) reduce the bioavailability of free IGF-1 [20,21,22,23]. This phenomenon, referred to as the “IGFBP trap,” results in functional IGF-1 deficiency despite normal or elevated total levels. Clinically, decreased IGF-1 bioavailability reflects impaired synthetic liver function and is associated with increased mortality risk, highlighting its value as a prognostic biomarker in both hepatic and renal disorders [24,25,26,27,28,29]. Figures were created using BioRender.com.

In pathological conditions, such as chronic kidney disease, increased IGFBP levels may reduce the availability of free, biologically active IGF-1—a phenomenon often referred to as the “IGFBP trap” [20,21,22,23] (Figure 3).

In transplantation, IGF-1 levels have been associated with graft function and patient survival. Reduced concentrations correlate with impaired regeneration and increased mortality, whereas higher early postoperative levels may indicate favorable graft function and outcomes [24,25,26,27].

### 2.4. Transforming β Growth Factor (TGF-β)

TGF-β is a multifunctional cytokine regulating immune responses, extracellular matrix formation, and tissue repair, with three major isoforms expressed in mammals [31,32,33]. It signals via SMAD-dependent pathways, influencing fibrosis, immune tolerance, and cellular differentiation [43,44].

**Figure 4:**
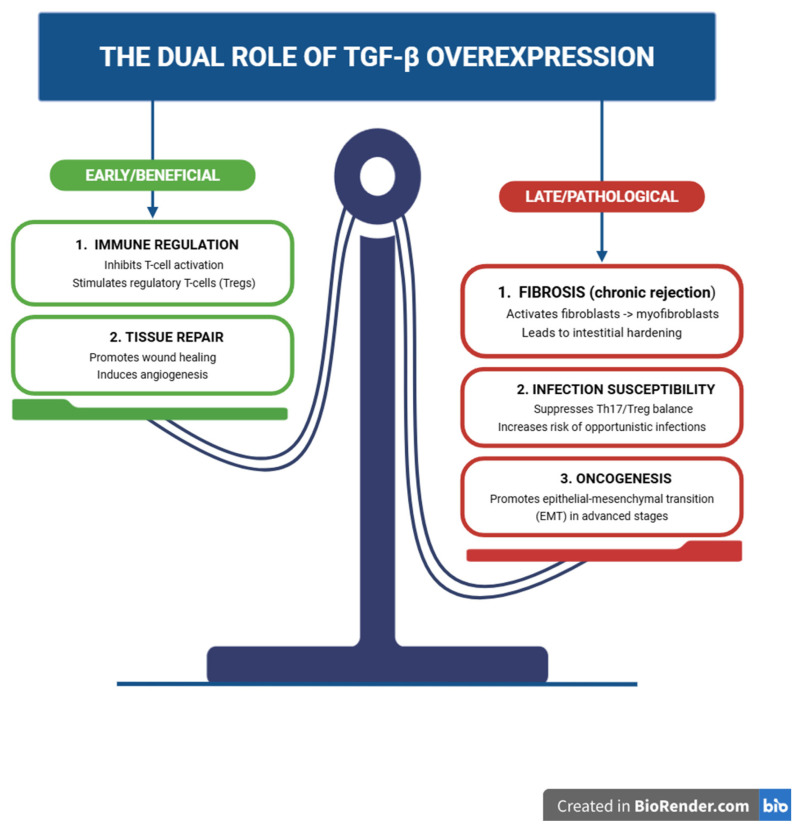
The context-dependent dual role of TGF-β signaling in transplantation. TGF-β exerts pleiotropic effects depending on temporal and microenvironmental context. In the early phase, it promotes immune tolerance, suppresses T-cell activation, and supports tissue repair and angiogenesis [31,32,43,44,45,46]. However, chronic overexpression drives pathological remodeling through fibroblast-to-myofibroblast transition, extracellular matrix accumulation, and sustained immunosuppression, increasing susceptibility to opportunistic infections [47,48,49,50,51,52,53,54]. In advanced stages, TGF-β contributes to oncogenesis through epithelial–mesenchymal transition (EMT) and tumor microenvironment remodeling [55]. Figures were created using BioRender.com.

Although TGF-β plays a role in early repair and immune regulation, its chronic activation promotes pathological fibrosis, graft remodeling, and immunosuppression [43,44]. This context-dependent dual role is summarized in Figure 4.

### 2.5. Vascular Endothelial Growth Factor (VEGF)

VEGF is a central regulator of angiogenesis, acting primarily through VEGFR-1 and VEGFR-2 to promote endothelial cell proliferation, migration, and vascular permeability [38]. Its expression increases under hypoxic and inflammatory conditions. In transplantation, VEGF plays a dual role: it supports early revascularization but may also contribute to endothelial dysfunction and chronic vascular complications when dysregulated [39,40,41,42].

## 3. Organ Transplantation

Organ transplantation remains one of the most effective treatments for end-stage organ failure; however, long-term outcomes are limited by both early and late post-transplant complications [56,57].

Post-transplant outcomes are influenced by multiple factors, including immune-mediated injury, infection, malignancy, and treatment-related toxicity [57,58,59].

**Figure 5:**
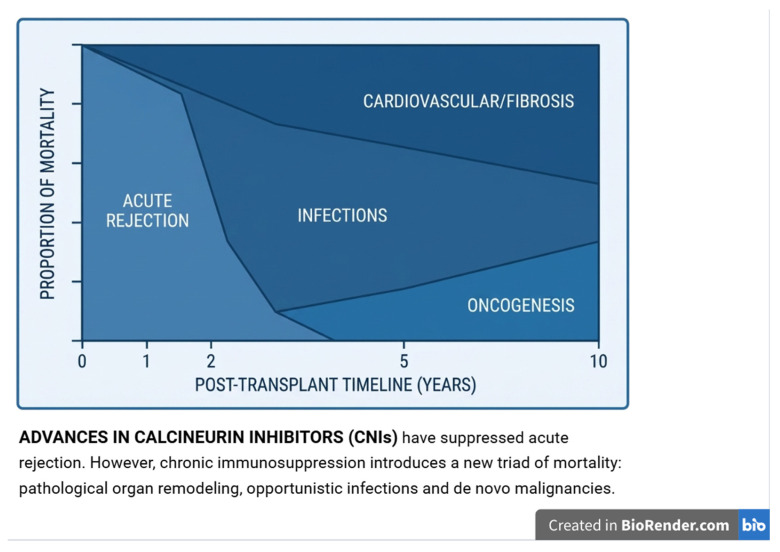
Conceptual timeline of post-transplant complications and mortality drivers. Advances in immunosuppressive therapy, particularly calcineurin inhibitors (CNIs), have significantly reduced the incidence of acute rejection in the early post-transplant period [57,63,64]. However, long-term outcomes are increasingly determined by chronic complications, including pathological organ remodeling (fibrosis and cardiovascular disease), opportunistic infections, and de novo malignancies [58,59,61,63,65,66]. This schematic illustrates the dynamic shift in dominant causes of morbidity and mortality over time, highlighting the transition from early immune-mediated injury to late-stage complications associated with prolonged immunosuppression [57,58,59,63,67]. Figures were created using BioRender.com.

The temporal evolution of these complications is illustrated in Figure 5. While advances in immunosuppressive therapy have significantly reduced the incidence of acute rejection, long-term outcomes are increasingly determined by chronic processes, including fibrosis, vascular remodeling, opportunistic infections, and malignancies [57].

These complications arise from complex interactions between ischemia-reperfusion injury, alloimmune responses, and prolonged immunosuppression, leading to persistent endothelial injury and tissue remodeling [56,57,58,59].

In this context, the limited sensitivity and specificity of currently used diagnostic markers remain a major clinical challenge, as complications are often detected only after irreversible damage has occurred [60,61].

Therefore, there is a critical need for early, mechanistically informative biomarkers that can improve risk stratification and predict long-term outcomes. Growth factors such as PDGF, TGF-β, VEGF, IGF-1, and EGF are increasingly recognized as key mediators linking tissue injury, immune activation, and graft remodeling [2,62].

## 4. Methodology

To evaluate the prognostic relevance of platelet-derived growth factors in solid organ transplantation, a structured literature search was conducted in the PubMed/MEDLINE database. The search included articles published up to December 2024 and was limited to English-language publications involving human subjects. No restrictions regarding study design were applied in the initial screening phase.

The following combinations of keywords were used:

“kidney transplantation” OR “renal transplant” OR “liver transplantation” OR “hepatic transplant” OR “heart transplantation” OR “cardiac transplant” combined with “PDGF”, “TGF-β”, “VEGF”, “IGF-1”, “EGF”, and “growth factors”. Additional searches included combinations of terms such as “survival”, “prognosis”, “mortality”, “fibrosis”, “vasculopathy”, and “infection risk” to identify studies specifically addressing clinical outcome prediction.

Titles and abstracts were screened for relevance to the role of growth factors as prognostic or predictive markers in post-transplant outcomes. Studies were included if they (1) evaluated circulating or tissue expression levels of PDGF, TGF-β, VEGF, IGF-1, or EGF in transplant recipients or donors, and (2) analyzed associations with clinically relevant endpoints such as graft survival, patient survival, opportunistic infections, primary graft dysfunction, or chronic allograft vasculopathy. Experimental animal studies were considered only if they provided mechanistic insight directly applicable to transplant pathology.

After removal of duplicates and exclusion of studies focusing exclusively on basic molecular biology without clinical correlation, 42 articles met the inclusion criteria and were subjected to full-text analysis. The most clinically relevant findings were synthesized and structured by transplanted organ type and biological pathway.

Given the heterogeneity of study designs, patient populations, sampling methods, and assay techniques, a formal meta-analysis was not feasible. Therefore, results were integrated using a qualitative comparative approach, with particular emphasis on reported cut-off values, hazard ratios, and survival associations.

Although this review does not follow a formal PRISMA framework, the search strategy was structured to enhance reproducibility and minimize selection bias.

This approach allows integration of heterogeneous clinical and mechanistic data to provide a comprehensive overview of growth factor–mediated processes in transplantation.

All figures were created by the authors using BioRender.com and do not involve AI-generated content requiring additional disclosure.

## 5. Growth Factors as Prognostic Markers of Survival After Organ Transplantation

### 5.1. Kidneys

Kidney transplantation remains the most effective treatment for end-stage renal disease, offering improved survival and quality of life compared with dialysis. Despite advances in surgical techniques and immunosuppressive therapy, long-term graft survival remains limited by chronic allograft dysfunction, which is the leading cause of graft loss [67]. Histologically, this condition is characterized by interstitial fibrosis and tubular atrophy, resulting from both immune- and non-immune mechanisms [68].

Growth factors such as TGF-β, PDGF, IGF-1, and VEGF play a central role in these processes by regulating fibrosis, angiogenesis, and extracellular matrix remodeling. Among them, TGF-β is considered an important mediator of fibrogenesis, promoting fibroblast-to-myofibroblast differentiation and collagen deposition, ultimately leading to progressive deterioration of graft function [69].

In the context of kidney disease and transplantation, dysregulation of IGF-1 and VEGF signaling has also been implicated in glomerular and vascular injury. Elevated levels of these factors are associated with endothelial dysfunction, increased vascular permeability, and progression of renal damage [19,69].

Given their involvement in key pathways of graft injury, growth factors may have potential as biomarkers for early detection of chronic graft dysfunction and for guiding immunosuppressive therapy. The following sections discuss their prognostic potential in kidney transplant recipients.

#### 5.1.1. Importance of PDGF in Kidney Transplantation

PDGF is one of the best-known fibrogenic growth factors involved in the development of many kidney diseases, including chronic transplant disease. Due to its structure, PDGF exists in several forms (PDGF-A, PDGF-B, PDGF-C, and PDGF-D) that differ in their biological properties, expression patterns, and receptor specificity. The role of specific PDGF isoforms in renal pathology, particularly in chronic graft rejection, is becoming clearer.

Increased expression of PDGF-A and PDGF-B has been observed in transplanted kidneys during vascular rejection, suggesting their involvement in vascular damage and in the recruitment of inflammatory cells. Particular attention is also paid to PDGF-D, as its overexpression leads to progressive glomerulonephritis. It has also been identified in patients with chronic graft nephropathy. In vivo studies have shown that an exogenous supply of PDGF-D induces intensive proliferation of mesangial cells, resulting in severe proliferative glomerulonephritis—an effect attributed to the strong mitogenic properties of this isoform [70]. Conversely, PDGF-C mediates the development of interstitial renal fibrosis, confirming its involvement in chronic damage to the transplanted organ [71,72].

Increased expression of the PDGFR-α receptor has been reported in kidney transplants that were rejected. Importantly, studies in animal models have shown that inhibiting PDGF signaling with imatinib, a PDGF receptor inhibitor, reduces lesions characteristic of chronic rejection and prevents graft fibrosis [69]. This suggests the potential for targeted therapy based on PDGF pathway blockade to be used alongside immunosuppressive therapy.

Immunosuppressive drugs, especially cyclosporine, also significantly affect PDGF expression. As early as 2002, Graff et al. [72] showed that cyclosporine use increases PDGF levels, which may promote fibrogenesis and accelerate graft damage. Compared to tacrolimus, cyclosporine induces greater platelet activation, leading to the release of growth factors such as PDGF and a fibrogenic effect on tubular-interstitial cells. Therefore, tacrolimus is now the more commonly used calcineurin inhibitor, with less effect on PDGF expression and a lower risk of chronic graft damage.

#### 5.1.2. Importance of TGF-β in Kidney Transplantation

TGF-β is one of the most important mediators of fibrosis in kidney pathology. Elevated levels of TGF-β are observed in patients awaiting a kidney transplant, particularly those undergoing chronic dialysis. The constant presence of vascular access and chronic inflammation induces excessive TGF-β expression, contributing to extracellular matrix deposition and the development of glomerular and interstitial fibrosis [73,74,75].

In kidney transplant recipients, TGF-β levels are higher than in healthy individuals and remain a significant risk factor for chronic graft nephropathy (CAN). TGF-β induces fibroblast proliferation and the transformation of tubular cells into myofibroblasts, resulting in tubular atrophy and interstitial fibrosis, which are typical features of chronic rejection [76,77].

A meta-analysis by Dosanjh et al. [78] showed a correlation between early TGF-β activation and increased fibrosis and impaired graft function after three years. The authors indicated that siRNA (small interfering RNA) therapies targeting TGF-β receptors may be a promising way to prevent the progression of fibrosis.

In acute graft rejection, increased expression of all three TGF-β isoforms was reported in the tubules and interstitium, with TGF-β1 predominating in the glomeruli. The involvement of TGF-β in leukocyte recruitment and inflammatory infiltrates suggests that its role extends beyond chronic fibrosis to include acute inflammatory mechanisms [76].

Interestingly, high levels of TGF-β are associated with both graft damage and increased susceptibility to opportunistic infections. Boix et al. [79] showed that TGF-β concentrations exceeding 808.51 pg/mL within the first six months after transplantation are associated with nearly double the risk of opportunistic infections in kidney and liver recipients. The researchers proposed that TGF-β could serve as a biomarker for both immunosuppression and infection risk during the post-transplant period.

Based on available data, TGF-β clearly plays a multifaceted, highly detrimental role in kidney transplantation. As a highly fibrogenic agent, TGF-β contributes to both chronic and acute graft damage, and it may also weaken the immune response, thereby increasing the risk of opportunistic infections.

Considering this evidence, TGF-β is a promising therapeutic target and diagnostic and prognostic biomarker for kidney transplant patients. Regular monitoring could facilitate early identification of rejection risk and infectious complications, enabling faster adjustments to immunosuppressive therapy. In the future, molecular therapies targeting the TGF-β pathway (e.g., siRNA, monoclonal antibodies) could also be used to prevent chronic rejection.

#### 5.1.3. Importance of IGF-1 in Kidney Transplantation

The somatotropic axis (GH/IGF-1 axis) plays a crucial role in the development and maintenance of kidney structure and function. GH and IGF-1 regulate renal cell regeneration, kidney mass, and filtration capacity, while renal function itself influences the activity of this hormonal axis. In patients with chronic kidney disease (CKD), IGF-1 bioavailability decreases despite normal or elevated total concentrations. This results from increased levels of insulin-like growth factor-binding proteins (IGFBPs), which are insufficiently cleared by the kidneys and additionally produced by inflammatory and hepatic cells, thereby limiting the amount of biologically active IGF-1 [19,29].

In patients undergoing renal replacement therapy, growth factor levels are variable. Cecerska-Heryć et al. [19] demonstrated that both the type and duration of therapy significantly affect IGF-1 and PDGF-B concentrations. Importantly, lower IGF-1 levels were associated with a significantly increased risk of mortality, highlighting its potential prognostic value.

In chronic allograft nephropathy (CAN), IGF-1 signaling is increased in moderate to advanced stages, whereas VEGF and PDGF pathways are more active in earlier phases of graft injury [78]. This suggests stage-dependent roles of growth factors and supports the concept of targeted modulation of IGF-1 signaling in advanced fibrosis.

IGF-1 also demonstrates therapeutic potential. Clinical studies indicate that exogenous IGF-1 administration may improve renal function in acute kidney injury and support recovery of filtration capacity after surgery [19]. These effects are mediated by anti-inflammatory activity, stimulation of cell proliferation, and improved renal perfusion. Overall, the GH/IGF-1 axis plays a dual role in renal pathophysiology: it supports regeneration and maintenance of kidney function, but its dysregulation contributes to disease progression. IGF-1 may therefore serve both as a prognostic biomarker and a potential therapeutic target in renal transplantation.

### 5.2. Liver

Liver transplantation is performed in patients with end-stage liver disease, most commonly due to cirrhosis (alcohol-related or viral, e.g., HBV/HCV), acute liver failure (e.g., paracetamol toxicity or autoimmune hepatitis), and hepatocellular carcinoma [80,81]. Despite advances in surgical techniques and immunosuppressive therapy, long-term outcomes remain limited by graft fibrosis, chronic rejection, and infectious complications.

In this context, growth factors such as IGF-1 and TGF-β play an important role in post-transplant adaptation and prognosis. IGF-1 is primarily synthesized by hepatocytes under the influence of growth hormone (GH), and its circulating levels reflect hepatic synthetic and metabolic function. In patients with liver failure, IGF-1 concentrations are significantly reduced, whereas normalization after transplantation reflects graft recovery and functional restoration. Persistently low IGF-1 levels after transplantation are associated with poorer prognosis and increased risk of complications or mortality [82,83].

TGF-β, in turn, exerts pleiotropic effects in the transplanted liver. While it contributes to tissue repair in the early post-transplant period, chronic activation promotes fibrogenesis and graft remodeling. Elevated TGF-β levels have been associated with an increased risk of graft fibrosis, chronic rejection, and biliary complications, including chronic cholangiopathy [83]. In addition, higher TGF-β concentrations have been linked to an increased risk of opportunistic infections in immunosuppressed patients [79].

#### 5.2.1. Importance of PDGF in Liver Transplantation

PDGF is a key mediator of liver fibrogenesis, with increased activity following organ injury. Among its isoforms, PDGF-B and PDGF-D are particularly relevant in chronic liver disease and transplantation, as they promote hepatic stellate cell (HSC) activation and proliferation. Activated HSCs differentiate into myofibroblasts and produce excessive extracellular matrix, leading to progressive fibrosis.

Increased expression of PDGF-B and PDGF-D has been observed in both acute and chronic liver injury. Similar findings have been reported in pediatric liver transplant recipients. Voutilainen et al. [84] demonstrated that children with graft fibrosis, even in the absence of inflammation, exhibited significantly higher expression of PDGF-α and PDGF-β genes compared with controls. These findings suggest that PDGF pathway activation may promote fibrosis independently of overt inflammatory responses [76,77]. In line with this, PDGF-D, a ligand for PDGFR-β, exerts strong mitogenic and fibrogenic effects, as shown in in vivo models demonstrating HSC activation and progressive fibrosis [85]. Increased expression of PDGF-B and PDGFR-β has also been reported in liver biopsies of patients with advanced cirrhosis, correlating with fibrosis severity [85].

Inhibition of PDGF signaling, for example, with tyrosine kinase inhibitors such as imatinib, reduces HSC activation, extracellular matrix deposition, and fibrosis in preclinical models [86]. These findings highlight PDGF as both a marker of fibrotic activity and a potential therapeutic target in liver transplantation.

Overall, PDGF signaling plays a central role in graft fibrosis, including forms that develop independently of active inflammation, suggesting that growth factor profiling may provide additional insight beyond traditional inflammatory markers in long-term graft monitoring.

#### 5.2.2. Importance of IGF-1 in Liver Transplantation

In liver diseases such as cirrhosis, disturbances of the GH/IGF-1 axis lead to reduced IGF-1 synthesis by hepatocytes. Decreased IGF-1 levels reflect impaired hepatic synthetic function and represent an independent prognostic factor, with lower concentrations associated with worse outcomes and increased mortality [87]. Studies by the Acute Liver Failure Study Group have further highlighted IGF-1’s potential as a biomarker of liver injury and regeneration, particularly in acute liver failure [88].

Following liver transplantation, rapid restoration of the GH/IGF-1 axis function is observed, accompanied by a significant increase in serum IGF-1 levels. As early as 15 days post-transplant, IGF-1 concentrations rise, reflecting recovery of graft synthetic function. IGF-1 levels exceeding 90 μg/L between days 15 and 30 have been shown to predict short-term survival (up to three months) with 86% sensitivity and 95% specificity (*p* < 0.001) [76]. These findings highlight the value of early IGF-1 assessment as a non-invasive indicator of graft quality and risk of complications (Table 2).

IGF-1 levels also correlate with standard biochemical markers of liver function, including bilirubin, albumin, and prothrombin. Nicollini et al. [89] demonstrated that IGF-1 may serve as a dynamic clinical indicator, reflecting changes in graft function and enabling earlier detection of dysfunction compared with conventional parameters. Thus, IGF-1 has both diagnostic and prognostic value in the post-transplant period.

#### 5.2.3. Importance of TGF-β in Liver Transplantation

TGF-β plays an important role in regenerative processes, fibrogenesis, and the regulation of the immune response following organ transplantation.

Boix et al. [77] have shown that elevated serum TGF-β levels correlate with an increased risk of opportunistic infections (OIs) in kidney and liver recipients. Analysis conducted between one and six months post-transplantation identified TGF-β threshold values with significant prognostic value. In liver transplant recipients, a TGF-β level greater than 363.25 pg/mL was associated with a 1.2-fold higher risk of OI, whereas in kidney recipients, the cut-off value was 808.51 pg/mL. The sensitivity and specificity of these parameters exceeded 70%, indicating their high clinical utility (Table 2).

The immunosuppressive effect of TGF-β may explain this relationship: this factor inhibits T lymphocyte activation and promotes the differentiation of regulatory lymphocytes (Tregs). It may also disrupt the balance between Th17 and Treg lymphocytes, affecting the immune response and increasing susceptibility to infection [92].

In light of the above data, TGF-β can be considered not only as a mediator of fibrosis but also as a potential biomarker for predicting the risk of opportunistic infections after transplantation. Regular monitoring of its concentration in the early postoperative period could enable the personalization of immunosuppressive treatment and the implementation of preventive measures in patients at higher risk.

The association between elevated TGF-β levels and an increased risk of opportunistic infections in organ recipients is an important addition to our understanding of TGF-β’s role in post-transplant immunoregulation. While TGF-β has primarily been associated with healing, regeneration, and fibrosis, an increasing number of studies, including that of Boix et al. [79], indicate that excessive TGF-β activity can suppress the immune response and increase the risk of infection.

These observations have practical implications: determining TGF-β levels in the early post-transplant period could inform extended patient monitoring and support therapeutic decisions. Incorporating this parameter into everyday clinical practice could enable the earlier detection of risk factors for infectious complications and the optimization of immunosuppression, particularly in patients for whom maintaining a balance between preventing rejection and minimizing infection risk is extremely difficult. Thus, TGF-β can serve as both an indicator of potential fibrosis progression and a predictor of infection risk, making it a particularly valuable biomarker in the care of organ recipients.

### 5.3. Heart

Heart transplantation is the final therapeutic option for patients with advanced heart failure when other treatments are no longer effective. Despite significant progress in surgical techniques and immunosuppressive therapy, long-term outcomes remain limited by early and late post-transplant complications [93].

Primary graft dysfunction (PGD) is the leading cause of early mortality after heart transplantation. It is primarily associated with ischemia-reperfusion injury, resulting in inflammatory activation, oxidative stress, and myocardial damage. PGD occurs in 2.5–30% of recipients, and severe forms are associated with high short-term mortality, reaching up to 54% within 30 days [93].

Early identification and management of PGD are critical. In severe cases, mechanical circulatory support, such as extracorporeal membrane oxygenation, may improve survival [94].

Given the central role of endothelial injury, inflammation, and vascular remodeling in both early graft dysfunction and long-term complications, growth factors—particularly VEGF—have emerged as important mediators and potential biomarkers in heart transplantation. Their role is discussed in detail in the following section.

#### 5.3.1. Importance of VEGF in Heart Transplantation

VEGF-A is increasingly evaluated in both transplant recipients and donors. Elevated donor VEGF-A levels have been associated with poorer early post-transplant outcomes. Holmström et al. [90] demonstrated that higher VEGF-A concentrations in donors correlate with increased myocardial injury in recipients, a higher incidence of primary graft dysfunction (PGD), prolonged intensive care unit stay, and greater need for circulatory and renal support.

VEGF-A also plays a significant role in long-term outcomes. Elevated levels have been linked to the development of cardiac allograft vasculopathy (CAV), a major limitation of long-term survival after heart transplantation. CAV is characterized by chronic endothelial injury and progressive intimal hyperplasia. Experimental studies suggest that VEGF signaling contributes to vascular remodeling, while VEGF receptor inhibition reduces atherosclerotic lesion formation [90].

Pediatric studies further indicate that VEGF-A levels above 90 pg/mL are an independent risk factor for moderate to severe CAV within five years post-transplantation [91]. These findings suggest that VEGF-A may serve not only as a mediator of vascular and inflammatory processes but also as an early prognostic biomarker.

In conclusion, assessing VEGF-A levels in cardiac donors may be a valuable approach for predicting both early complications, such as PGD, and long-term outcomes, including CAV. Incorporating this parameter into donor evaluation could improve risk stratification and transplant outcomes (Table 2). It should be noted that some reported cut-off values are derived from experimental or ex vivo stimulation models rather than direct serum measurements, which may limit their immediate clinical applicability and warrant cautious interpretation.

#### 5.3.2. Importance of PDGF in Heart Transplantation

PDGF expression is significantly increased in biopsies in both acute and chronic vascular rejection. All samples from hearts exhibiting global ischemia expressed PDGF, whereas biopsies from hearts without the condition expressed PDGF in only 44%. Studies in rats have shown that PDGF-A impacts the development of coronary artery inner membrane hypertrophy in a chronic rejection model following heart transplantation. A cause-and-effect relationship has been demonstrated between PDGF-A and this event, and it has been proven that blocking the PDGF-A receptor significantly attenuates this process [95].

The study by Tuuminem et al. [96] indicated that treatment with PDGFR-α antibodies, rather than PDGF-A antibodies alone, reduced the development of graft atherosclerosis. This also suggests a role for PDGF-C in the development of this condition, as it binds the same receptor. The alloimmune response induced the expression of PDGF-A, PDGF-C, and PDGF-D in the transplanted heart’s vessels. Overexpression of these ligands led to increased TGF-β1 production and accelerated fibrosis and arteriosclerosis in the transplanted heart. In contrast, PDGF-B did not exhibit this effect, indicating that its involvement in chronic rejection is less significant. Overall, this study suggests that inhibiting all PDGF isoforms except PDGF-B may effectively prevent chronic rejection in heart transplants.

Furthermore, studies in animal models have demonstrated that the overexpression of PDGF-C and PDGF-D accelerates the development of cardiac fibrosis and arteriosclerosis. Notably, PDGF-D overexpression led to proliferation of interstitial fibroblasts, extensive collagen deposition, and arterial wall thickening, thereby promoting cardiac fibrosis and atherosclerosis [95].

Overall, both experimental and clinical data clearly indicate that PDGF, particularly the PDGF-A, PDGF-C, and PDGF-D isoforms, plays a significant role in the development of vascular lesions in transplanted hearts. Blocking the relevant receptors could be a promising way to prevent chronic vascular allopathy after heart transplantation.

#### 5.3.3. Importance of TGF-β in Heart Transplantation

Research by Gichkun et al. [97] suggests that TGF-β1 may play a role in the development of graft rejection and fibrosis following heart transplantation. Patients with end-stage heart failure had significantly higher plasma concentrations of TGF-β1 than the control group. Following heart transplantation, the level of this factor decreased significantly compared with the preoperative value and remained low for up to 1 year. The authors suggest that a lack of decrease in TGF-β1 concentration after surgery may indicate an increased risk of adverse postoperative events [97].

While no significant correlation was observed between TGF-β1 levels and myocardial fibrosis, a significant relationship was observed between TGF-β1 concentration and the degree of mismatch within the HLA system. Higher levels of TGF-β1 were detected in patients with a higher percentage of mismatched HLA antigens, suggesting its involvement in immune mechanisms leading to rejection. However, as the authors emphasize, this relationship requires further in-depth research [97].

In a review published in Nature Reviews Nephrology, Meng et al. [98] detailed the central role of TGF-β1 as a major regulator of fibrosis processes in various organs, including the heart and kidneys. According to the authors, TGF-β1 has a multifaceted effect: it activates fibroblasts, induces epithelial-mesenchymal transition, and increases the synthesis of extracellular matrix proteins, including types I and III collagen. Chronic overactivation of the TGF-β1 pathway has been shown to cause irreversible structural changes in tissues, contributing to fibrosis and organ failure.

Similarly, Sureshbabu et al. [99] described the complex mechanisms through which TGF-β1 influences inflammation and fibrosis in kidney disease. They also noted the possibility of translating these mechanisms to other organs, including the heart. They noted that TGF-β1 activates fibroblasts and inhibits the immune response, which may explain its dual role as both a protector and a pathogen after transplantation. Research suggests that modulating TGF-β1 or TGF-β1 receptor expression could be an effective therapeutic strategy for preventing chronic graft damage.

Conversely, Ren et al. [100] conducted a comprehensive analysis of the molecular pathways underlying TGF-β1-mediated organ fibrosis. The article emphasizes that the TGF-β1 pathway primarily activates Smad2/3, which then combine with transcriptional coactivators to regulate the expression of pro-inflammatory and profibrotic genes. The authors also noted that TGF-β1 may interact with other signaling pathways, such as MAPK or PI3K/Akt, thereby enhancing its effects under oxidative stress or ischemia-reperfusion, which are often present after heart transplantation.

Extending the analyses of Gichkun et al. [97] with data from more recent studies [98,99,100] confirms that TGF-β1 is not only a biomarker of rejection but also plays an active role in the structural and immune changes that occur in the transplanted heart. Its effect is highly context-dependent: it can support immune tolerance, but chronic activation leads to fibrosis and loss of organ function. The decrease in TGF-β1 levels observed in Gichkun’s studies after transplantation may therefore indicate a reduction in profibrotic and pro-inflammatory stimuli. Conversely, its persistence at a high level may indicate ongoing pathological remodeling. Consequently, TGF-β1 could serve not only as an indicator of clinical status but also as a therapeutic target, as evidenced by the increasing number of preclinical studies on inhibitors of this pathway.

### 5.4. Lung

Lung transplantation is the final therapeutic option for patients with end-stage lung diseases, including chronic obstructive pulmonary disease (COPD), idiopathic pulmonary fibrosis, cystic fibrosis, and pulmonary arterial hypertension. Despite significant advances in surgical techniques and immunosuppressive therapy, lung transplantation remains associated with high rates of early and late complications compared to other solid organ transplants.

One of the most critical early complications is primary graft dysfunction (PGD), which occurs within the first 72 h after transplantation and is a major cause of early morbidity and mortality. PGD is primarily driven by ischemia-reperfusion injury (IRI), leading to endothelial damage, increased vascular permeability, pulmonary edema, and activation of inflammatory cascades. These processes are accompanied by oxidative stress and recruitment of innate immune cells, which further amplify tissue injury and impair graft function [101].

In the long term, chronic lung allograft dysfunction (CLAD), particularly bronchiolitis obliterans syndrome (BOS), represents the main limitation to survival after lung transplantation. BOS is characterized by progressive airway fibrosis, luminal narrowing, and irreversible decline in lung function. The pathogenesis of BOS involves persistent inflammation, immune activation, and dysregulated tissue remodeling, all of which are closely linked to dysregulated growth factor signaling [102].

Growth factors such as VEGF and TGF-β appear to play a particularly important role in lung transplant pathology. VEGF contributes to increased vascular permeability and pulmonary edema during ischemia-reperfusion injury, while also regulating endothelial repair processes. However, excessive VEGF signaling may exacerbate inflammatory responses and worsen primary graft dysfunction. In contrast, TGF-β is a key mediator of fibroproliferative remodeling and has been strongly implicated in the development of bronchiolitis obliterans syndrome, promoting fibroblast activation and extracellular matrix deposition. These observations suggest that dysregulated growth factor signaling may directly contribute to both early graft injury and long-term airway fibrosis in lung transplant recipients [102,103,104,105].

This further supports the concept of growth factors as integrative mediators of immune-vascular-stromal crosstalk and potential modulators of both early graft injury and long-term remodeling across solid organ transplantation.

Importantly, early dysregulation of VEGF and TGF-β signaling during ischemia-reperfusion injury may improve risk stratification and determine the trajectory toward either graft recovery or progression to chronic lung allograft dysfunction, highlighting their potential as early predictive biomarkers rather than late-stage indicators of damage.

#### Importance of VEGF and TGF-β in Lung Transplantation

Growth factors such as VEGF and TGF-β play a significant role in both early and late complications after lung transplantation. VEGF contributes to increased vascular permeability and edema formation during IRI, while also regulating angiogenesis and endothelial repair. However, excessive VEGF signaling may exacerbate inflammatory responses and vascular leakage, thereby contributing to PGD severity [101,102].

TGF-β, in turn, is a key mediator of fibroproliferative remodeling and has been strongly implicated in the development of BOS. Elevated TGF-β signaling promotes fibroblast activation, extracellular matrix deposition, and airway fibrosis, leading to progressive graft dysfunction. These findings suggest that dysregulated growth factor signaling may play a central role in both acute injury and chronic remodeling of the transplanted lung [102,103].

Although less extensively studied than in kidney, liver, or heart transplantation, the available evidence indicates that growth factor-mediated pathways in lung transplantation reflect similar mechanisms of immune activation, endothelial injury, and tissue remodeling. Therefore, lung transplantation further supports the concept that growth factor profiling may provide valuable insights into graft adaptation and long-term outcomes across different organ systems.

## 6. Limitations of Current Evidence and Challenges for Clinical Translation

Although platelet-derived growth factors show promising associations with graft dysfunction and survival, several limitations hinder their immediate clinical implementation.

An additional limitation concerns the central concept of the platelet activation axis itself. Although several of the discussed mediators are stored in platelet α-granules, the current literature does not provide sufficient direct evidence that persistent platelet activation is the dominant source of sustained growth factor elevation in transplant recipients.

To date, and to the best of our knowledge, no clinical studies have assessed platelet activation markers—such as P-selectin, PF4, and β-thromboglobulin—alongside circulating growth factor levels and long-term transplant outcomes in the same patient cohorts. Consequently, it remains unclear whether elevated levels of PDGF, TGF-β, VEGF, or EGF are primarily due to sustained platelet activation or to non-platelet cellular sources, including macrophages, endothelial cells, and fibroblasts.

This limitation reduces the specificity of growth factors as surrogate markers of platelet activity and underscores the need for integrated biomarker approaches combining platelet activation markers with growth factor profiling. Consequently, the platelet activation axis should be interpreted as a conceptual and hypothesis-generating framework rather than a fully validated mechanistic model. Most available data derive from observational, predominantly single-center studies with small cohorts [79,90,97]. Proposed prognostic cut-off values—such as TGF-β thresholds for opportunistic infection risk or early postoperative IGF-1 levels predicting short-term survival—have not been extensively validated in large, multicenter prospective trials [79,89]. Furthermore, the incremental prognostic value of growth factors beyond established markers—such as donor-specific antibodies (DSA) [56], inflammatory markers, or transcriptomic biopsy profiling [106]—remains insufficiently defined.

Methodological variability represents a major challenge. Since PDGF, TGF-β, and VEGF are stored in platelet α-granules [5,8], ex vivo platelet activation during blood collection and processing may significantly influence measured concentrations. Differences in serum and plasma measurements, preanalytical handling, and sampling timing can substantially affect results, limiting cross-study comparability. Standardized sampling and processing protocols are therefore essential before clinical integration.

Causality also remains uncertain. While TGF-β is widely recognized as a central mediator of fibrosis [98,99,100], and PDGF signaling contributes to vascular remodeling and chronic rejection [71,96], these mediators may also reflect compensatory repair mechanisms rather than primary drivers of pathology. Similarly, VEGF exhibits both protective and deleterious effects depending on ischemic and inflammatory context [39,90]. This biological duality complicates therapeutic targeting.

Finally, growth factors rarely act in isolation. Their integration into multiparametric prognostic models incorporating immunological, inflammatory, and molecular markers has not yet been systematically evaluated. Advanced modeling strategies—including Cox regression-based survival analysis and integrative risk stratification—are needed to determine whether growth factor profiling independently predicts long-term mortality and graft failure.

Addressing these limitations through standardized methodologies, prospective validation, and integrative modeling will be essential to translate the platelet activation axis from a mechanistic concept into a clinically actionable prognostic tool. Accordingly, current evidence should be interpreted as hypothesis-generating rather than sufficient for immediate clinical implementation.

## 7. Contribution of Innate Immune Cells to Growth Factor Signaling and Graft Remodeling

In addition to platelets, innate immune cells—particularly monocytes and macrophages—play a central role in graft injury, chronic rejection, and tissue remodeling after solid organ transplantation. These cells represent a major non-platelet source of several growth factors discussed in this review, including TGF-β, PDGF, and VEGF, and contribute to their sustained expression within the graft microenvironment [31,32,33,34,35,38,39,40,49,50,51,52,53,54].

Monocyte-derived macrophages infiltrate transplanted tissues early after ischemia-reperfusion injury and remain active during chronic phases of graft remodeling. They contribute to extracellular matrix deposition, angiogenesis, and vascular remodeling, particularly through the production of TGF-β and PDGF, which promote interstitial fibrosis and chronic graft dysfunction [49,50,51,52,53,54]. In addition, macrophage–endothelial interactions may amplify VEGF-mediated angiogenic signaling and vascular permeability within the graft microenvironment [38,39,40].

Recent advances in spatial transcriptomics have provided further insight into the cellular sources of growth factor signaling in transplant pathology. A study by Wongworawat et al. (2025), using 10x Visium spatial transcriptomics in kidney allograft biopsies, identified FCGR3A^+^ monocyte/macrophage subpopulations that co-localize with rejection lesions and are enriched in pathways associated with TGF-β and PDGF signaling [107]. These findings support the role of macrophages as key contributors to growth factor-driven graft remodeling. Similar observations in experimental and clinical studies indicate that macrophage activation is a major driver of fibrosis and chronic allograft injury across multiple organ systems [49,50,51,52,53,54].

These findings highlight that circulating and tissue-level growth factor profiles reflect a complex interplay between platelets and immune cells rather than a platelet-exclusive process. Consequently, the platelet activation axis should be interpreted within a broader framework of immune-vascular-stromal crosstalk, in which platelet activation is one of several interacting components driving graft adaptation and injury. Growth factor levels should therefore be interpreted as a composite signal reflecting systemic inflammation, tissue injury, and activation of multiple cellular compartments, rather than a single dominant source.

## 8. Clinical Implementation Framework: Integrating the Platelet Activation Axis into Post-Transplant Care

To translate the platelet activation axis into clinical practice, a structured implementation strategy is required.

First, growth factor assessment should be incorporated into predefined postoperative time windows rather than performed as isolated measurements. Early-phase evaluation (e.g., within the first 1–6 months) may identify patients at increased risk of opportunistic infections (TGF-β) [79] or impaired early graft recovery (IGF-1) [89]. In later phases, persistent elevation of PDGF or VEGF may indicate progressive vascular remodeling or chronic allograft vasculopathy [90,96].

Second, growth factor profiling should be interpreted within a multiparametric framework. Rather than replacing established diagnostics such as DSA monitoring [56], inflammatory markers, or transcriptomic signatures [106], platelet-derived mediators may complement these tools by providing mechanistic insight into remodeling and survival risk. Integration into composite risk scores could improve early identification of patients requiring intensified surveillance or tailored immunosuppressive adjustments.

Third, standardized preanalytical protocols are mandatory to minimize variability related to platelet activation during sampling [5,8]. Harmonization of assay methods and establishment of organ-specific reference ranges will be critical steps toward reproducibility.

Finally, longitudinal monitoring—rather than single time-point assessment—may better capture dynamic remodeling processes. The trajectory of growth factor changes over time could prove more informative for survival prediction than absolute concentrations alone.

By embedding growth factor profiling within structured, multiparametric risk models and standardized laboratory workflows, the platelet activation axis may evolve from a mechanistic hypothesis into a practical component of precision transplant medicine.

## 9. Conclusions

Growth factors represent central regulators of tissue homeostasis, immune modulation, and vascular remodeling. In the context of solid organ transplantation, their dysregulation reflects the complex interplay between ischemia-reperfusion injury, alloimmune activation, chronic immunosuppression, and progressive graft remodeling. Emerging evidence suggests that alterations in their circulating levels are not merely epiphenomena of tissue injury but may serve as clinically meaningful indicators of long-term outcomes.

Importantly, these mediators are unlikely to provide sufficient predictive accuracy as standalone biomarkers and should be interpreted within integrated multiparametric models combining immunological, inflammatory, and molecular data.

Among the growth factors discussed, TGF-β has shown potential as a surrogate marker of risk for opportunistic infections. However, its circulating levels likely reflect the combined activity of platelets, immune cells, and tissue remodeling compartments. PDGF is strongly implicated in interstitial fibrosis and vascular rejection across kidney, liver, and heart transplantation, and its expression may be influenced by calcineurin inhibitor toxicity, suggesting potential utility in monitoring treatment-related remodeling. IGF-1 reflects hepatic synthetic function and regenerative capacity. At the same time, low systemic levels are associated with increased mortality in advanced renal disease, positioning it as both a prognostic indicator and potential therapeutic target. VEGF-A, particularly when assessed in both donor and recipient, correlates with cardiac allograft vasculopathy and post-transplant mortality, underscoring its predictive relevance in heart transplantation. Although EGF has primarily been investigated in oncology, its role in epithelial regeneration and malignancy risk warrants further evaluation in chronically immunosuppressed transplant populations.

Importantly, longitudinal assessment of growth factor dynamics may enable more accurate risk stratification than single-time-point measurements. Such an approach could support individualized adjustment of immunosuppressive regimens, including decisions to minimize calcineurin inhibitors, introduce mTOR inhibitors, or intensify surveillance in patients at elevated risk of fibrosis, infection, or malignancy.

Future studies should focus on integrating growth factor profiling with direct markers of platelet activation and immune cell dynamics, and on validating these approaches in large, prospective, multicenter cohorts. Ultimately, embedding growth factor trajectories into multiparametric clinical models may contribute to the development of precision transplant strategies that better balance immune suppression, graft protection, and long-term survival.

## Figures and Tables

**Table 1:** Characteristics of selected growth factors involved in post-transplant pathology. Platelet activation represents one of several contributing mechanisms rather than a dominant source of growth factor release.

Growth Factor	Biological Function	Role in Transplantation	References
PDGF	Mesenchymal cell proliferation, fibroblast activation, and extracellular matrix deposition	Promotes interstitial fibrosis, vascular remodeling, and chronic graft dysfunction	[9,10,11,12,13]
TGF-β	Immune modulation, fibroblast-to-myofibroblast transition, and collagen synthesis	Central mediator of fibrosis; dual role in tissue repair and chronic rejection	[30,31,32,33,34,35]
EGF	Epithelial proliferation, tissue repair, and cell survival signaling	May contribute to epithelial regeneration; dysregulation linked to inflammation and tumorigenesis	[36,37]
VEGF	Angiogenesis, endothelial cell proliferation, vascular permeability	Supports revascularization but contributes to endothelial dysfunction and vasculopathy	[38,39,40,41,42]
IGF-1	Cell growth, metabolism, and anti-apoptotic signaling	Reflects graft function (especially liver); associated with survival and regeneration	[17,18,19,20,24,25,26,27,28,29]

**Table 2:** Proposed prognostic cut-off values for growth factors in organ transplantation.

Organ	Biomarker	Cut-Off Value/Condition	Sample/Matrix	Clinical Significance (Outcome Prediction)
Kidney	TGF-β	>808.51 pg/mL (first 6 months)	Plasma (ex vivo stimulation) *	Associated with nearly a 2-fold higher risk of opportunistic infections [79].
Liver	TGF-β	>363.25 pg/mL (first 6 months)	Plasma (ex vivo stimulation) *	Associated with a 1.2-fold higher risk of opportunistic infections [79].
Liver	IGF-1	>90 µg/L (days 15–30 post-tx)	Serum	Predictor of short-term survival (3 months) with 86% sensitivity and 95% specificity [89].
Heart	VEGF-A (Recipient)	>90 pg/mL	Serum/Plasma **	Independent risk factor for moderate to severe CAV within 5 years (pediatric) [90].
Heart	VEGF-A (Donor)	Elevated levels	Donor serum	Associated with increased myocardial injury and incidence of PGD in recipients [91].

Footnote: * Values derived from activation-dependent measurements of TGF-β rather than direct circulating levels. ** Sample type was not consistently specified in the original study.

## Data Availability

No new data were created or analyzed in this study. Data sharing is not applicable to this article.
